# Impact of rhomboid intercostal plane block on chronic pain incidence and acute pain scores after oncologic breast surgery: a randomized controlled trial

**DOI:** 10.1186/s12871-026-04175-2

**Published:** 2026-08-12

**Authors:** Tolga Karaçay, Basak Altiparmak, Sinan Pektas, Cenk Yazkan, Bakiye Ugur

**Affiliations:** 1 Department of Anesthesiology and Reanimation, Mugla Sitki Koçman University, Mugla, Turkey; 2 Department of General Surgery, Mugla Sitki Koçman University, Mugla, Turkey

**Keywords:** Acute pain, Chronic pain, Postoperative complications, Regional anesthesia, Rhomboid intercostal plane block

## Abstract

**Background:**

Persistent chronic pain after oncologic breast surgery remains a major clinical problem, affecting approximately 25–60% of patients and significantly impairing quality of life. Although the rhomboid intercostal plane block (RIB) has been shown to reduce acute postoperative pain, its role in preventing chronic postoperative pain remains inadequately investigated.

**Methods:**

This prospective, single-center, randomized, double-blind, controlled trial included 254 adult women undergoing oncologic breast surgery with or without lymph node dissection. Patients were randomized to receive either a pre-operative ultrasound-guided single-shot RIB using 30 mL of 0.25% bupivacaine or no regional block (control group). All patients received standardized multimodal systemic analgesia. The primary outcome was the incidence of chronic pain at 3 months postoperatively. Secondary outcomes included chronic pain at 6 months, Brief Pain Inventory–Short Form scores at 3 and 6 months, neuropathic pain assessed using the Douleur Neuropathique 4 questionnaire, acute postoperative pain scores, and 24-h postoperative opioid consumption.

**Results:**

Of the 254 randomized patients, 247 completed the study. Chronic pain incidence was significantly lower in the RIB group at 3 months (17% vs 31%; *p* = 0.009) and 6 months (13% vs 30%; *p* < 0.001), with reduced neuropathic pain at 6 months. The RIB group had lower 24-h opioid consumption than controls *(p* = 0.01).

**Conclusions:**

Pre-operative single-shot RIB significantly reduced chronic postoperative pain and opioid consumption following oncologic breast surgery, and may be considered as part of multimodal analgesia to decrease chronic pain incidence, neuropathic pain and improve acute postoperative pain control.

**Trial registration:**

Clinicaltrials.gov (NCT06019325 registered August 17, 2023).

**Supplementary Information:**

The online version contains supplementary material available at https://doi.org/10.1186/s12871-026-04175-2.

## Background

Breast cancer is the most frequently diagnosed cancer in women worldwide [1]. Treatment typically involves a multidisciplinary approach including surgery, chemotherapy, radiotherapy, and adjuvant therapies [2]. Despite advances in treatments, persistent postoperative pain remains a common and debilitating complication, reported in 25% to 60% of patients [3]. This condition is typically defined as pain affecting the breast, axillary region, or ipsilateral arm that persists beyond 3 months after surgery [4]. Identified risk factors include younger age, axillary lymph node dissection (ALND), adjuvant therapies, psychological vulnerability, and poorly controlled acute postoperative pain [5–7]. Inadequate perioperative analgesia may not only impair early recovery through delayed mobilization and respiratory compromise, but also contribute to the transition from acute to persistent pain [8].

Post-mastectomy pain is anatomically complex and arises from injury to multiple tissue planes and neural structures, including the pectoral fascia, axillary dissection sites, and the anterior and lateral cutaneous branches of the intercostal nerves (T2–T6), as well as the intercostobrachial nerve. In addition, traction or injury to the pectoral, long thoracic, and thoracodorsal nerves may contribute to persistent nociceptive and neuropathic pain components [9].

In recent years, regional anesthesia techniques such as paravertebral block (PVB), serratus anterior plane block (SAPB), erector spinae plane block (ESPB), and pectoral blocks have been increasingly used for postoperative analgesia following oncologic breast surgery [5]. However, variability in dermatomal spread and proximity to the surgical field have led to continued interest in alternative interfascial plane approaches. The rhomboid intercostal plane block (RIB) has been described as a potential alternative, providing analgesia by blocking the lateral cutaneous branches of the T3–T9 intercostal nerves [10]. Nevertheless, its impact on persistent postoperative pain has not been well established. The RIB may offer certain practical and anatomical features that make it suitable for evaluation in this setting. The injection site is anatomically separate from the primary surgical field, which may facilitate perioperative workflow. In addition, ultrasound visualization of the relevant anatomical landmarks is typically feasible, potentially supporting procedural consistency. These considerations provided the rationale for evaluating RIB in the present trial.

This prospective, randomized, double-blind, controlled study aimed to evaluate the effect of pre-operative RIB with 0.25% bupivacaine on acute and persistent pain following oncologic breast surgery with or without ALND. The primary hypothesis was that RIB would reduce the incidence of persistent postoperative pain at 3 months, defined by a Brief Pain Inventory–Short Form (BPI-SF) item 5 score = 3. Secondary hypotheses were that, compared with the control group, patients receiving RIB would have a lower incidence of chronic pain at 6 months, lower BPI-SF scores, lower incidence of neuropathic pain, lower acute postoperative pain scores, reduced postoperative nausea and vomiting, and decreased 24-h opioid consumption.

## Methods

### Study population and regulatory aspects

This prospective, single-center, randomized, double-blind controlled trial was conducted in accordance with the Consolidated Standards of Reporting Trials (CONSORT) guidelines at Mugla Sitki Koçman University. Ethical approval was obtained from the Scientific Research and Ethics Committee (Chair: Prof. Dr. Ümmühani Özel Türkçü; 23 March 2023; Decision No: 7/X). This study was prospectively registered at ClinicalTrials.gov (NCT06019325, First submitted: August 17, 2023; First posted: August 31, 2023; Last update posted: September 24, 2025). The first participant was enrolled on September 7, 2023, and data collection was completed on April 15, 2025. All procedures involving human participants were conducted in accordance with the ethical standards of the institutional research committee and with the principles of the Declaration of the World Medical Association (Declaration of Helsinki, 2024 revision). Written informed consent was obtained from all participants before enrollment.

Women aged 18–70 years with ASA physical status I–III undergoing elective oncologic breast surgery (modified radical mastectomy (MRM), simple mastectomy, or partial mastectomy) with or without ALND were eligible. Exclusion criteria included chronic opioid use, substance or alcohol abuse, analgesic use within 24 h preoperatively, BMI > 35 kg/m2, local infection at the injection site, hypersensitivity to local anesthetics, planned bilateral mastectomy, anticipated surgery duration > 180 min, or expected postoperative communication difficulties.

### Study design

Patients were randomized in a 1:1 ratio using a web-based computer-generated randomization system. Allocation concealment was ensured using sequentially numbered, opaque, sealed envelopes prepared by an independent researcher not involved in patient recruitment or outcome assessment. Envelopes were opened after induction of general anesthesia by the attending anesthesiologist. Patients and postoperative assessors were blinded to group allocation. To maintain blinding, RIB was performed after the induction of general anesthesia.

### Anesthesia management

Standard monitoring was applied to all patients, including non-invasive blood pressure, electrocardiography, and pulse oximetry. General anesthesia was induced and maintained using standard intravenous (IV) agents with thiopental (5 mg/kg), fentanyl (2 µg/kg), and rocuronium bromide (0.6 mg/kg).

### Interventions

**Fig. 1:**
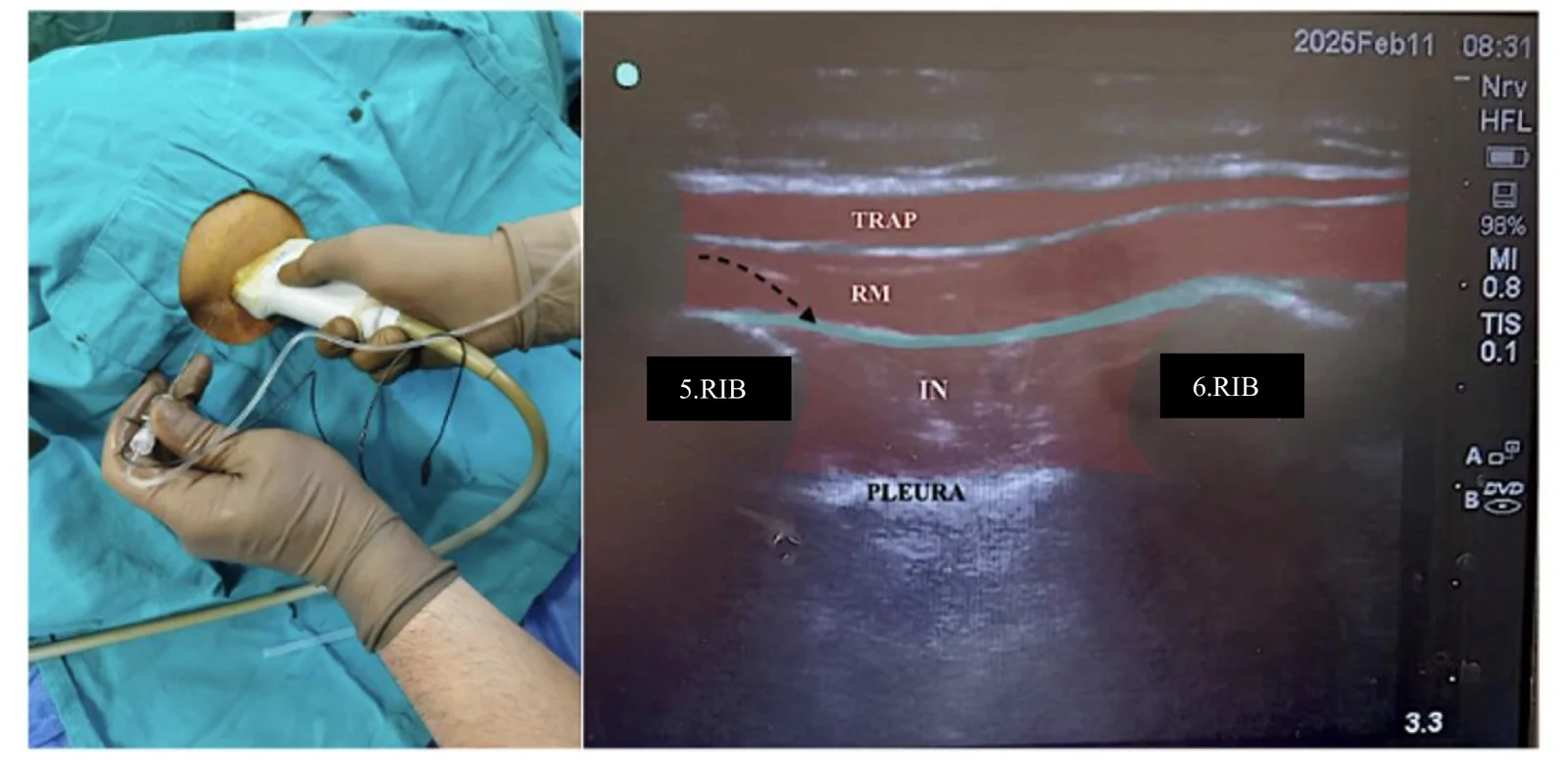
Ultrasound-guided technique of the rhomboid intercostal plane block. IN, intercostal muscles; RM, rhomboid muscle; TRAP, trapezius muscle

After endotracheal intubation, patients were positioned laterally. In the RIB group, a high-frequency linear ultrasound probe (6–13 MHz, Sonosite M-Turbo, Fujifilm Inc.) was placed in an oblique sagittal plane near the medial scapular border at the T5–T6 level. Following sterile preparation, the fascial plane between the rhomboid major and intercostal muscles was identified, and 30 mL of 0.25% bupivacaine was injected after confirmation of the correct interfascial plane by hydrodissection with normal saline (Fig. 1). All RIB procedures were performed by anesthesiologists with more than 10 years of experience in ultrasound-guided regional anesthesia and specific experience with the RIB technique. The total dose of local anesthetic did not exceed 2 mg/kg, which is the recommended maximum single-dose limit for bupivacaine in adult regional anesthesia practice as described in standard anesthetic pharmacology references. No regional block was performed in the control group. All patients received standardized intraoperative multimodal analgesia consisting of IV dexketoprofen 50 mg and tramadol 1 mg/kg administered 15 min before the end of surgery, along with ondansetron 4 mg for antiemetic prophylaxis.

### Postoperative pain assessment and outcomes

Postoperative pain was assessed using the Numerical Rating Scale (NRS; 0 = no pain, 10 = worst imaginable pain). In the recovery unit, pain was assessed at 15 and 30 min using the NRS. Patients with an NRS score = 4 at the 15-min assessment received IV fentanyl (1 µg/kg). The NRS was reassessed at 30 min to evaluate analgesic response. Additional fentanyl was not routinely administered at the 30-min assessment if fentanyl had already been given at 15 min. In the surgical ward, patients received IV paracetamol 1000 mg three times daily and dexketoprofen 50 mg twice daily. Pain was evaluated at 1, 2, 6, 12, and 24 h postoperatively. Rescue analgesia was defined as administration of intravenous tramadol 50 mg when NRS = 4. Total 24-h opioid consumption was recorded.

At 3 and 6 months postoperatively, patients were evaluated by a pain specialist using the Brief Pain Inventory–Short Form (BPI-SF) and the Douleur Neuropathique 4 (DN4) questionnaire. These instruments are validated tools that have been previously published and widely used in clinical research. Higher scores in BPI-SF and DN4 indicate worse pain intensity and greater neuropathic pain components, respectively, whereas lower scores indicate better pain control and lower symptom burden [11, 12]. Patients were specifically asked whether the pain was located in the operated breast and/or axillary region. In cases where patients did not attend these visits, repeated telephone calls were made to invite them, and their evaluations were completed within one week. BPI-SF item 5 was used to assess average pain over the past 24 h. A score = 3 at 3 months was defined as chronic postoperative pain. The total BPI-SF score (0–70 or 0–120 depending on scaling) was used only for overall pain burden assessment. Neuropathic pain was defined as a DN4 score = 4.

The primary outcome was the incidence of chronic pain at 3 months after surgery. The BPI-SF is a multidimensional tool measuring pain intensity and its interference with seven aspects of daily life (sleep, general activity, mood, work, social relationships, walking, and enjoyment), with total scores ranging from 0 to 120. Secondary outcomes included total BPI-SF scores, incidence of neuropathic pain at 3 and 6 months, acute postoperative pain assessed using the NRS at 15 and 30 min and at 1, 2, 6, 12, and 24 h, postoperative nausea and vomiting, and total 24-h opioid consumption.

### Sample size calculation and statistical analysis

Sample size calculation was performed using G*Power 3.1 software (Faul, Erdfelder, Buchner, & Lang, 2009). It was based on the incidence of chronic postoperative pain at 3 months after breast surgery reported in reference [3] (40% vs 23%). With a two-sided a of 0.05 and 80% power, 115 patients per group were required. Allowing for a 10% dropout rate, 254 patients were enrolled.

Mean and standard deviations or median and Q1 and Q3 values were presented for describing continuous variables. Frequency and percentages were used for presenting categorical variables. Continuous variables were analyzed using parametric T-test, mixed effects models or nonparametric test for longitudinal data (which is implemented in R's nparLD package). Categorical variables were compared using the chi-square or Fisher’s exact test accordingly. Post hoc comparisons were based on least squares means or nonparametric posthoc tests where appropriate. Statistical analyses were performed using SPSS software package version 25 (SPSS Inc.) and R version 4.4.2(R Core Team, 2024). Statistical significance was set at *P* < 0.05.

## Results

No interim analyses or stopping guidelines were planned. The trial protocol remained unchanged throughout the study, and no pre-specified subgroup or sensitivity analyses were performed. The trial was completed as planned without early termination, and patients or the public were not involved in the design, conduct, reporting, or dissemination of the research.

**Fig. 2:**
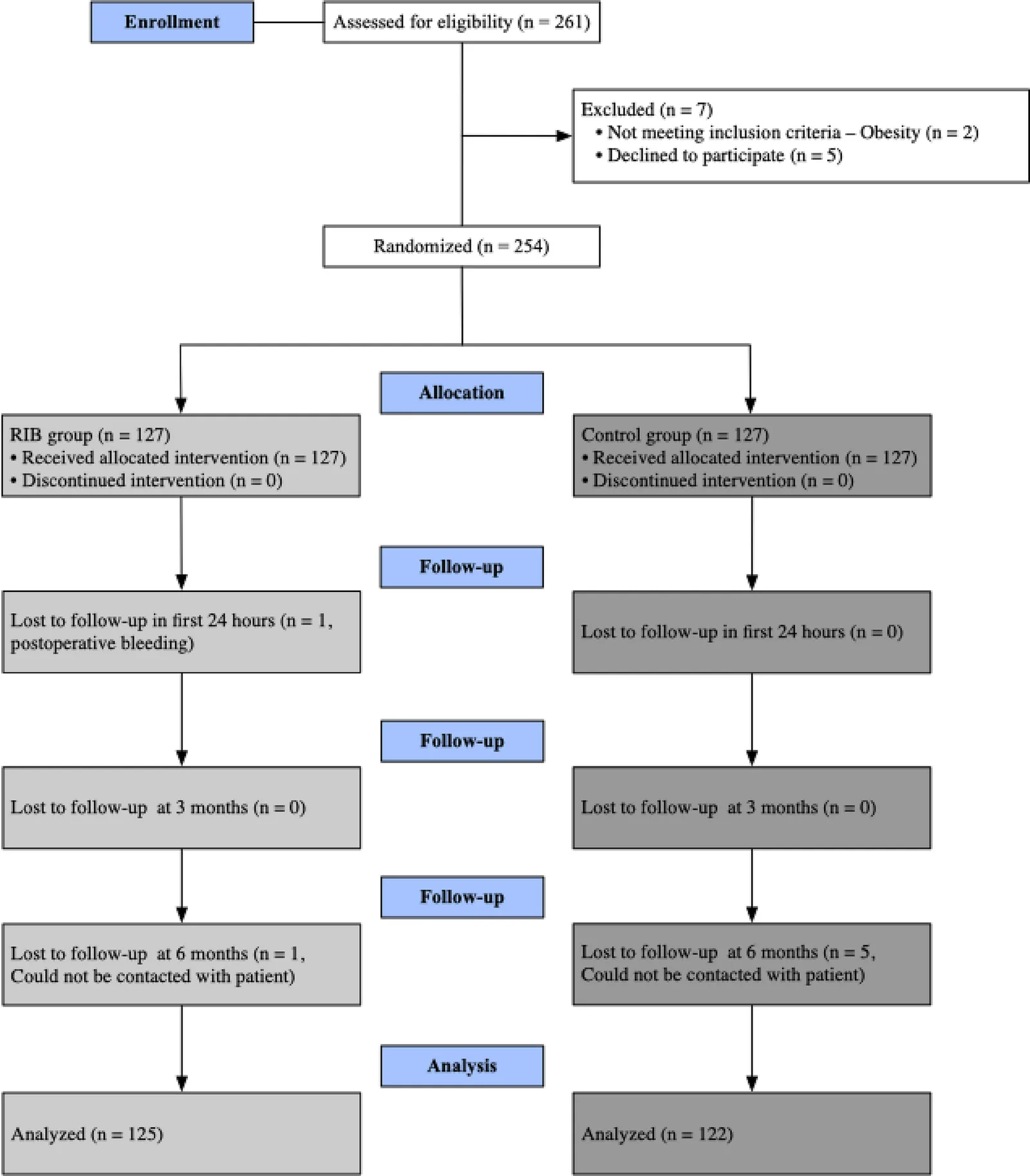
Flowchart, RIB, rhomboid intercostal plane block

A total of 261 patients were screened for eligibility beginning on 7 September 2023. Two patients were excluded due to obesity, and five declined participation. Consequently, 254 patients were randomized. One patient in the RIB group was excluded due to postoperative hemorrhage, leaving 253 patients available for postoperative and 3-month analyses. Between the 3- and 6-month follow-up assessments, an additional five patients in the control group and one patient in the RIB group were lost to follow-up. Therefore, the 6-month analysis was completed with 247 patients (Fig. 2).

Baseline demographic and clinical characteristics were comparable between groups. No significant differences were observed in age, height, weight, BMI, ASA physical status, type of surgery, history of chemoradiotherapy, postoperative chemoradiotherapy, or ALND (*p* > 0.05) (Table 1).

**Table 1:** Demographic and clinical characteristics of the groups

Variable	Control Group (*n* = 127)	RIB Group (*n* = 126)	p*
Age	55,13	53,12	0,11
Height(cm)	162,01 ± 5,98	161,50 ± 5,27	0,47
Weight(kg)	71,57 ± 11,37	71,01 ± 11,39	0,7
BMI	27,29 ± 4,16	27,20 ± 4,12	0,86
ASA			0,625
I	8 (%6,29)	12 (9,52)	
II	112 (%88,18)	108 (%85,71)	
III	7 (%5,51)	6 (%4,76)	
Type of Surgery			0,38
Mastectomy	40 (%31,50)	52 (%41,27)	
BCS	66 (%51,97)	56 (%44,44)	
BCS + ALND	6 (%4,72)	7 (%5,56)	
MRM	15 (%11,81)	11 (%8,73)	
Chemoradiotherapy	102 (%80,3)	103 (%81,7)	0,768

Values are presented as mean ± SD or *n* (%). Control group, patients without regional block; RIB group, patients receiving rhomboid intercostal plane block

*ALND* Axillary lymph node dissection, *BMI* Body mass index, *BCS* Breast-conserving surgery, *MRM* Modified radical mastectomy, *RIB* Rhomboid intercostal plane block

^*^p values were calculated using the chi-square test or Student’s t-test, as appropriate

### Chronic pain outcomes

At 3 months, mean BPI-SF item 5 scores did not differ significantly between groups (*p* = 0.35). In contrast, at 6 months, mean scores were significantly lower in the RIB group (*p* = 0.001). Total BPI-SF scores at 6 months were also significantly reduced in the RIB group compared with controls (6.85 ± 12.60 vs. 12.52 ± 15.34; *p* = 0.002).

**Fig. 3:**
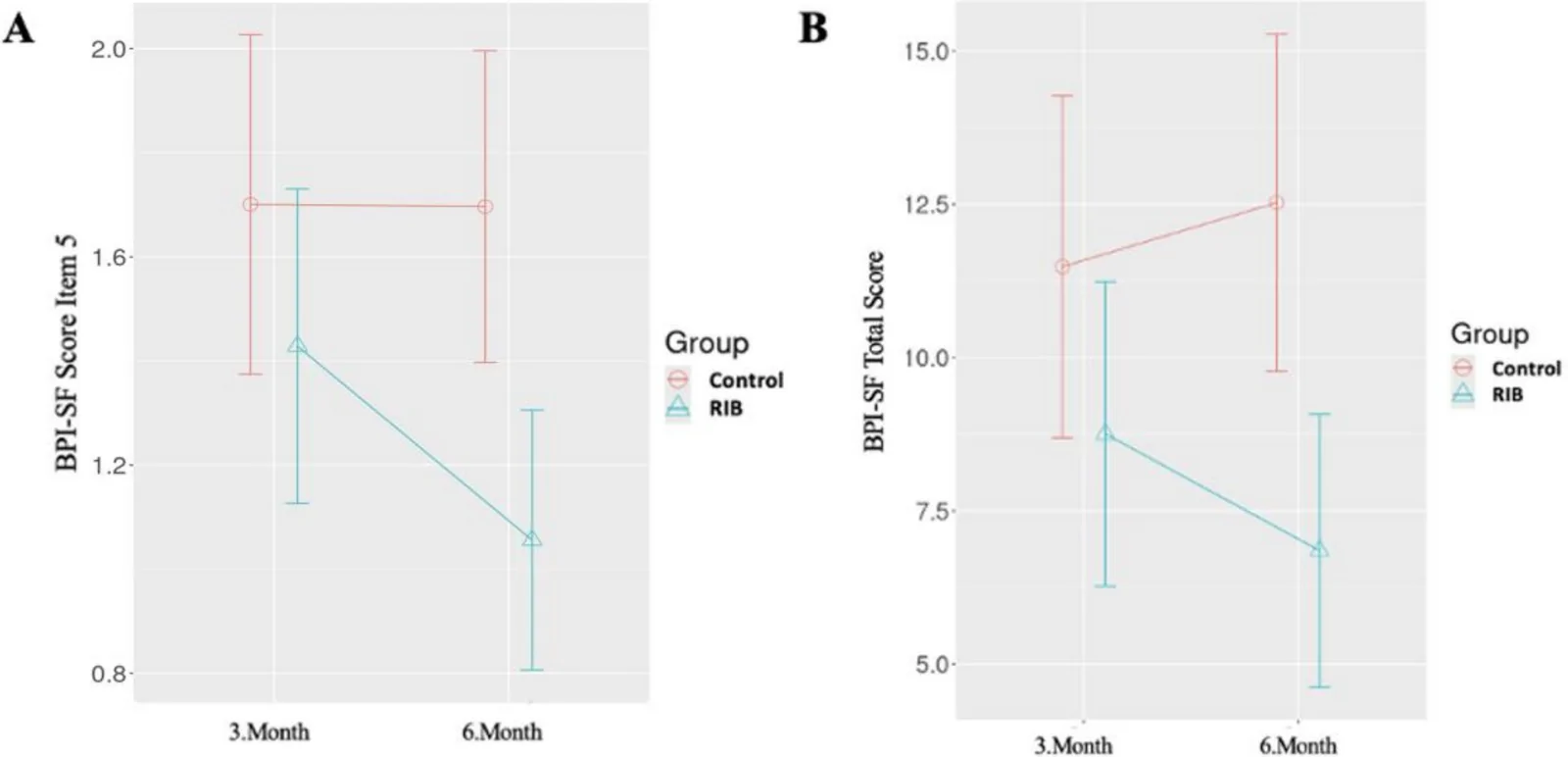
**A** BPI-SF score item 5, (**B**) BPI-SF total score. BPI-SF, brief pain inventory short form; RIB, rhomboid intercostal plane block. Values are expressed as mean ± 95% confidence interval (95% CI)

When chronic pain was defined as BPI-SF item 5 = 3, its incidence was significantly lower in the RIB group at both follow-up points. At 3 months, persistent pain was present in 17% (*n* = 22) of patients in the RIB group versus 31% (*n* = 40) in the control group (*p* = 0.009). At 6 months, chronic pain persisted in 13% (*n* = 16) of patients in the RIB group compared with 30% (*n* = 37) in the control group (*p* < 0.001) (Tables 2, 3 and Fig. 3).

**Table 2:** BPI-SF item 5 scores and chronic pain incidence of the groups

BPI-SF	Control Group, (*n* = 127)	RIB Group, (*n* = 126)	p*
Item 5. 3.month	1,70 ± 1,86	1,43 ± 1,71	0,35
Item 5. 3.month			
Pain present	40 (%31)	22 (%17)	**0,009**

BPI-SF	Control Group, (*n* = 122)	RIB Group, (*n* = 125)	p*
Item 5. 6.month	1,70 ± 1,67	1,06 ± 1,41	**0,001**
Item 5. 6.month			
Pain present	37 (%30)	16 (%13)	**< 0,001**

Values are presented as mean ± SD or *n* (%). Control group, patients without regional block; RIB group, patients receiving rhomboid intercostal plane block

*BPI-SF* Brief Pain Inventory–Short Form, *RIB* Rhomboid intercostal plane block

^*^p values for BPI-SF Item 5 were calculated using a nonparametric test

**Table 3:** BPI-SF total scores of the groups

BPI-SF	Control Group,	RIB Group,	p*
Total score 3. month (*n* = 127/*n* = 126)	11,48 ± 15,90	8,75 ± 14,07	0,14
Total score 6. month (*n* = 122/*n* = 125)	12,52 ± 15,34	6,85 ± 12,60	**0,002**

Values are presented as mean ± SD. Control group, patients without regional block; RIB group, patients receiving rhomboid intercostal plane block

*BPI-SF* Brief Pain Inventory–Short Form, *RIB* Rhomboid intercostal plane block

^*^p values for total BPI-SF scores were calculated using a mixed-effects model

Mean DN4 scores were comparable between groups at 3 months (*p* = 0.08) but were significantly lower in the RIB group at 6 months (*p* = 0.023). The incidence of neuropathic pain (DN4 = 4) followed a similar pattern. At 3 months, neuropathic pain was observed in 36% of the RIB group and 47% of controls (*p* = 0.063). At 6 months, these rates were 30% and 50%, respectively (*p* = 0.002) (Table 4).

**Table 4:** DN4 scores of the groups

DN4	Control Group, (*n* = 127)	RIB Group, (*n* = 126)	p*
3. month score	3,54 ± 2,19	3,08 ± 2,13	0,08
3. month			0,063
Pain present	60 (%47)	45 (%36)	

	Control Group, (*n* = 122)	RIB Group, (*n* = 125)	
6. month score	3,44 ± 2,42	2,75 ± 2,12	**0,023**
6. month			**0,002**
Pain present	61 (%50)	38 (%30)	

Values are presented as mean ± SD or n (%). Control group, patients without regional block; RIB group, patients receiving rhomboid intercostal plane block

*DN4* Douleur Neuropathique 4, *RIB* Rhomboid intercostal plane block

^*^p values were calculated using nonparametric test for longitudinal data and chi-square test

### Acute postoperative outcomes

At 24 h, 15.75% of patients in the control group and 5.56% in the RIB group required rescue tramadol (*p* = 0.01). The average total opioid consumption within 24 h was significantly lower in the RIB group compared with the control group (23.41 ± 9.8 mg vs. 37.01 ± 12.3 mg, respectively; *p* < 0.001) (Table 5).

**Table 5:** Opioid consumption of the group

Opioid	Control Group (*n* = 127)	RIB Group (*n* = 126)	p*
15. min			0,67
Fentanyl	29 (%22,83)	26 (%20,63)	
30. min			0,49
Fentanyl	11 (%8,66)	8 (%6,35)	
1. hours			0,91
Tramadol	33 (%25,98)	32 (%25,40)	
2. hours			0,052
Tramadol	13 (%10,24)	5 (%3,97)	
6. hours			0,11
Tramadol	18 (%14,17)	10 (%7,94)	
12. hours			0,28
Tramadol	9 (%7,09)	5 (%3,97)	
24. hours			**0,01**
Tramadol	20 (%15,75)	7 (%5,56)	
Total Opioid Consumption Dose(mg)	37.01 ± 12,3	23.41 ± 9,8	**< 0.001**

Values are presented as *n* (%) or mean ± SD. Control group, patients without regional block; RIB group, patients receiving rhomboid intercostal plane block

*RIB* Rhomboid intercostal plane block

^*^p values were calculated using the chi-square test or Student’s t-test, as appropriate

Postoperative NRS pain scores were numerically lower in the RIB group throughout the first 24 h. Statistically significant differences were observed at 15 min (*p* = 0.026) and 24 h (*p* = 0.002); however, the magnitude of these differences did not reach clinical significance. No significant differences were found between groups in the incidence of postoperative nausea and vomiting at any time point (*p* > 0.05) (Table 6).

**Table 6:** NRS scores of the groups

NRS	Control Group, (*n* = 127)	RIB Group (*n* = 126)	p*
15. min	2.00 (0.00—3.00)	2.00(0.00–3.00)	**0,026**
30. min	2.00 (0.00—3.00)	2.00(0.00–3.00)	0,061
1. hours	3.00(2.00–4.00)	2.00(1.00–4.00)	0,222
2. hours	2.00(1.00–3.00)	2.00(1.00–3.00)	0,454
6. hours	2.00(1.00–3.00)	2.00(1.00–2.00)	0,274
12. hours	1.00(0.00–2.00)	1.00(0.00–2.00)	0,663
24. hours	2.00(0.00–3.00)	1.00(0.00–2.00)	**0,002**

Values are presented as median (Q1-Q3). Control group, patients without regional block; RIB group, patients receiving rhomboid intercostal plane block

*NRS* Numeric rating scale, *RIB* Rhomboid intercostal plane block

^*^p values were calculated using the nonparametric test for longitudinal data

## Discussion

The principal finding of this study is that ultrasound-guided RIB, when added to general anesthesia for oncologic breast surgery, significantly reduced the incidence of chronic postoperative pain at both 3 and 6 months, while also decreasing postoperative opioid consumption. These findings suggest that RIB may provide not only effective acute analgesia but also contribute to reducing the transition from acute postoperative pain to persistent pain.

Initially described in 2016 by Elsharkawy et al. [10], RIB is thought to provide hemithoracic analgesia by blocking the lateral cutaneous branches of intercostal nerves from T3 to T9. Several studies have reported its analgesic efficacy in mastectomy, rib fracture, and thoracic surgery, including reductions in opioid consumption [13–15]. The first randomized controlled trial assessing RIB in breast surgery was published by Altiparmak et al. [16], in 2020, demonstrating improved quality of recovery scores and reduced opioid consumption within the first 24 h compared with controls.

The RIB targets the fascial plane between the rhomboid major and intercostal muscles, allowing spread of local anesthetic to the lateral cutaneous branches of multiple thoracic intercostal nerves, thereby providing multi-dermatomal analgesia. By attenuating afferent nociceptive input, RIB may reduce central sensitization processes involved in postoperative pain chronification [9].

Consistent with these mechanisms and the findings of Altiparmak et al., our study confirms effective acute analgesia and further extends the literature by demonstrating its potential association with reduced chronic post-mastectomy pain, an outcome not previously evaluated in randomized controlled trials.

Persistent post-surgical pain after breast surgery has been associated with poorly controlled acute pain, adjuvant therapies, surgical duration, and type of surgical procedure [17]. In this context, adequate perioperative analgesia is considered crucial in reducing central sensitization and the development of chronic pain. Although PVB remains one of the most established regional anesthesia techniques in breast surgery and is recommended in current guidelines, evidence regarding its long-term effects on persistent postoperative pain is still limited. Randomized trials by Karmakar et al. [18] and Qian et al. [19] reported lower rates of persistent pain at 3 and 6 months following PVB. Albi-Feldzer et al. [20] similarly reported reduced persistent pain; however, the authors acknowledged methodological limitations, including the use of the Visual Analog Scale, which may not fully capture the multidimensional nature of chronic pain.

Our findings were consistent with these results, as the RIB group demonstrated significantly lower chronic pain incidence at both 3 and 6 months. Furthermore, BPI-SF item 5 scores were significantly lower in the RIB group compared with controls at 6 months (*p* = 0.001), further confirming the long-term pain reduction benefits of RIB. Evidence regarding the effect of interfascial plane blocks on chronic postoperative pain after breast surgery remains limited and heterogeneous. Fujii et al. [21] reported lower chronic pain rates with both pecto-serratus and SAPB, whereas De Cassai et al. [4] observed a benefit only at 3 months. Similarly, Sulak et al. [22] found improved acute analgesia after SAPB but no reduction in chronic or neuropathic pain. Studies evaluating ESPB have also reported inconsistent findings, with some demonstrating reduced chronic pain compared with controls, while others found no long-term benefit [23–25]. These discrepancies may reflect differences in block technique, dermatomal spread, surgical procedures, follow-up duration, and pain assessment methods. In contrast to several previous studies relying mainly on NRS or LANSS, we used the multidimensional BPI-SF, which assesses both pain intensity and functional interference.

Studies specifically evaluating neuropathic pain after breast surgery remain scarce. Previous investigations assessing paravertebral or interfascial plane blocks have generally reported no significant reduction in neuropathic pain despite improvements in acute analgesia [20, 22, 25]. In our study, DN4 scores were comparable at 3 months; however, both the incidence of neuropathic pain and mean DN4 scores were significantly lower in the RIB group at 6 months. It is plausible that improved early postoperative analgesia reduced sustained nociceptive input, thereby attenuating central sensitization processes. Additionally, although primarily exerting local effects, local anesthetics have been suggested to possess systemic anti-inflammatory and antihyperalgesic properties, which may contribute to modulation of neuropathic pain pathways. Nevertheless, these mechanistic explanations remain speculative and warrant further investigation [26, 27].

Numerous studies have investigated secondary outcomes; acute pain, opioid consumption, and nausea/vomiting after breast surgery. Jiang et al. [28] compared RIB, ESP, and SAP blocks in MRM and found no significant differences in opioid use and pain scores. However, all blocks reduced 24-h tramadol use and postoperative pain. Çiftçi et al. [29] evaluated pectoserratus, RIB, and control groups in breast conserving surgery and reported lower acute pain and opioid use in block groups. Gökçen et al. [30] compared RIB, pectoral blocks, and local infiltration, finding improved quality of life, pain scores, and reduced opioid use with RIB. Finally, Chen et al.’s [31] meta-analysis of eight RCTs (*n* = 714) found that RIB significantly reduced 24-h pain scores, opioid consumption, and nausea/vomiting incidence. Our results mirrored the literature: significantly lower acute pain scores at 15 min and 24 h in the RIB group (*p* = 0.026 and *p* = 0.002, respectively), though the differences lacked clinical significance; significantly lower 24-h and total opioid consumption in the RIB group (*p* < 0.001); and no between-group differences in nausea/vomiting incidence.

To our knowledge, this randomized controlled trial is among the first to evaluate the effect of RIB on persistent pain after oncologic breast surgery. Strengths of the study include its face-to-face assessment of chronic pain outcomes using two validated and complementary instruments (BPI-SF and DN4). The sample size was adequately powered to detect clinically relevant differences in chronic pain incidence.

Our study has some limitations. First, follow-up was limited to 6 months. Although a six-month follow-up period is generally considered sufficient for the diagnosis of chronic pain, longer-term follow-up of our patients may provide additional data and further insights in the future. Second; as all blocks were performed after the induction of general anesthesia, it was not possible to assess dermatomal sensory blockade before surgery. In addition, to preserve patient blinding, postoperative dermatomal assessment was not performed, either. However, we believe that the likelihood of block failure, and consequently study dropout related to unsuccessful blocks, was low.

## Conclusions

In this single-center randomized controlled trial, preoperative ultrasound-guided RIB was associated with a lower incidence of persistent postoperative pain at 3 and 6 months following oncologic breast surgery. A reduction in pain severity and neuropathic pain measures became evident at 6 months. In addition, RIB improved selected early postoperative pain scores and reduced 24-h opioid consumption. Although primarily used for acute analgesia, these findings suggest that RIB may also influence longer-term pain outcomes. However, further multicenter studies are warranted to confirm these observations.

## Supplementary Information

Supplementary Material 1.

Supplementary Material 2.

Supplementary Material 3.

## Data Availability

Data are available by contacting the corresponding author through email.

## Abbreviations

ALND: Axillary Lymph Node Dissection
ASA: American Society of Anesthesiologists
BMI: Body Mass Index
BPI-SF: Brief Pain Inventory–Short Form
CONSORT: Consolidated Standards of Reporting Trials
DN4: Douleur Neuropathique 4 questionnaire
ESPB: Erector Spinae Plane Block
IV: Intravenous
LANSS: Leeds Assessment of Neuropathic Symptoms and Signs
MRM: Modified Radical Mastectomy
NRS: Numerical Rating Scale
PVB: Paravertebral Block
RIB: Rhomboid Intercostal Plane Block
SAPB: Serratus Anterior Plane Block
