# Supplementary material for: Impact of rhomboid intercostal plane block on chronic pain incidence and acute pain scores after oncologic breast surgery: a randomized controlled trial
Source: BMC Anesthesiol. 2026 Aug 12;26:662. doi: 10.1186/s12871-026-04175-2 (PMC13746700; doi:10.1186/s12871-026-04175-2)
Supplement: Supplementary file 1 [file 12871_2026_4175_MOESM1_ESM.pdf]

Patient Trial No	| |	| |

BRIEF PAIN INVENTORY (SHORT FORM)

Patient Initials:
Date of Birth(dd,mm,yyyy):
Visit Date (dd,mm,yyyy) :

1. Throughout our lives, most of us have had pain from time to time (such as minor headaches, sprains, and toothaches). Have you had pain other than these everyday kinds of pain during the last week?

 Yes No

2. On the diagram, shade in the areas where you feel pain. Put an X on the area that hurts the most.

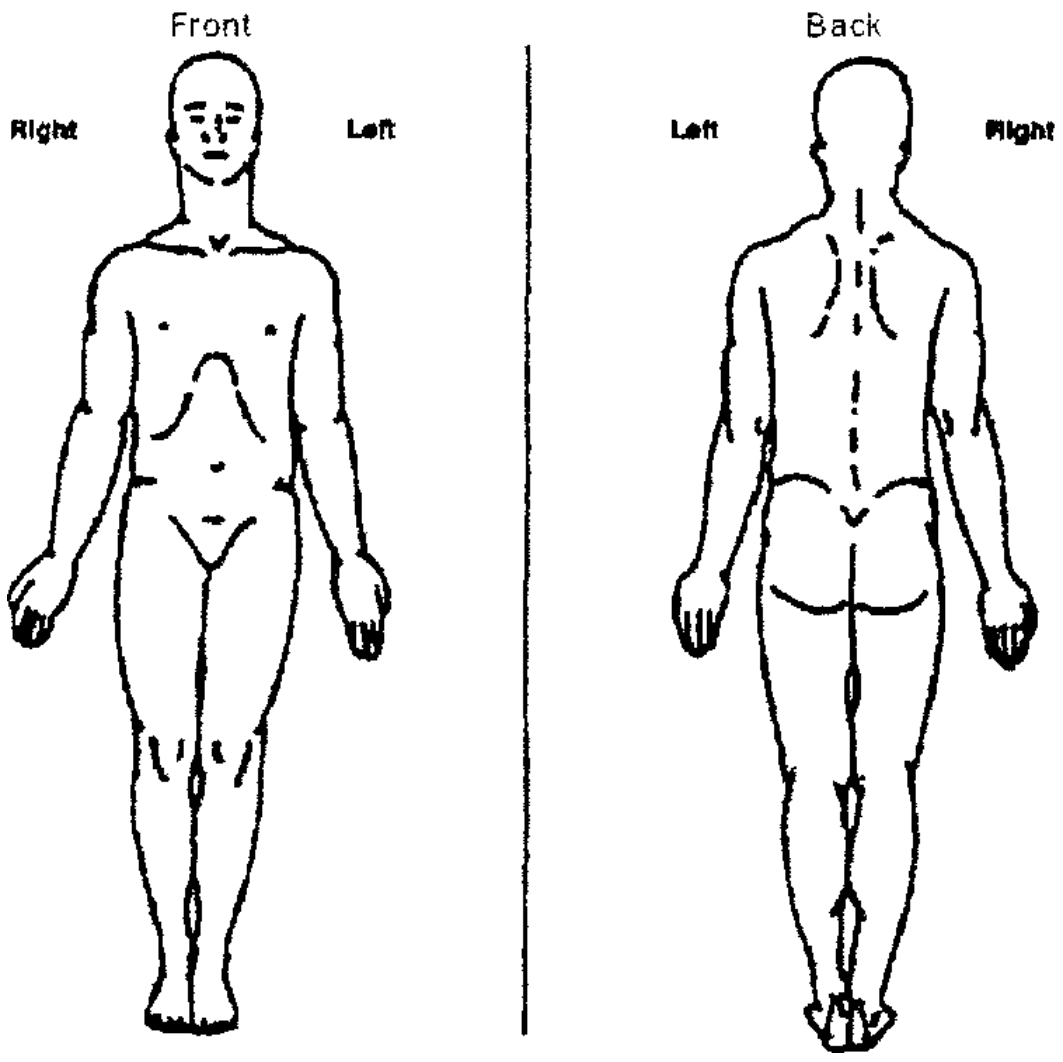

3. Please rate your pain by circling the one number that best describes your pain at its ***worst*** in the last week.

0	1	2	3	4	5	6	7	8	9	10
No Pain									Pain as bad as you can imagine	

4. Please rate your pain by circling the one number that best describes your pain at its ***least*** in the last week.

0	1	2	3	4	5	6	7	8	9	10
No Pain									Pain as bad as you can imagine	

5. Please rate your pain by circling the one number that best describes your pain on the ***average****.*

0	1	2	3	4	5	6	7	8	9	10
No Pain									Pain as bad as you can imagine	

6. Please rate your pain by circling the one number that tells how much pain you have ***right now****.*

0	1	2	3	4	5	6	7	8	9	10
No Pain									Pain as bad as you can imagine	

7.What treatments or medications are you receiving for your pain?

____ _

8. In the last week, how much relief have pain treatments or medications provided? Please circle the one percentage that most shows how much ***relief*** *you* have received.

0%	10%	20%	30%	40%	50%	60%	70%	80%	90%	100%
No Relief										Complete Relief

9. Circle the one number that describes how much, during the past week, pain has interfered with your:

A. General Activity

0	1	2	3	4	5	6	7	8	9	10
Does not Interfere										Completely Interferes

B. Mood

0	1	2	3	4	5	6	7	8	9	10
Does not Interfere										Completely Interferes

C. Walking Ability

0	1	2	3	4	5	6	7	8	9	10
Does not Interfere										Completely Interferes

D. Normal Work (includes both work outside the home and housework)

0	1	2	3	4	5	6	7	8	9	10
Does not Interfere										Completely Interferes

E. Relations with other people

0	1	2	3	4	5	6	7	8	9	10
Does not Interfere										Completely Interferes

F. Sleep

0	1	2	3	4	5	6	7	8	9	10
Does not Interfere										Completely Interferes

G. Enjoyment of life

0	1	2	3	4	5	6	7	8	9	10
Does not Interfere										Completely Interferes

Scoring:

Pain Severity Score = Mean of items 3-6 (pain at its worst, pain at its least, average Pain Interference Score = Mean of items 9A-9G (interference of pain with: general activity, mood, walking, normal work, relations, sleep, enjoyment of life)
