# Supplementary material for: Impact of rhomboid intercostal plane block on chronic pain incidence and acute pain scores after oncologic breast surgery: a randomized controlled trial
Source: BMC Anesthesiol. 2026 Aug 12;26:662. doi: 10.1186/s12871-026-04175-2 (PMC13746700; doi:10.1186/s12871-026-04175-2)
Supplement: Supplementary file 2 [file 12871_2026_4175_MOESM3_ESM.pdf]

Table 1 *Douleur Neuropathique 4 Questions* (DN4)

Neuropathic pain DN4 Questionnaire

Answer the four questions below with Yes or No for each item:	
Patient Interview	
Question1: dose your pain present one or more of the following characteristics	
1.Pain feels like burning<br>2. Sensation of painful cold<br>3. Pain feels like electric shocks	
Question 2: in the same area, is your pain associated to one or more symptoms?	
4. Tingling<br>5. Pins and needles<br>6. Numbness<br>7. Itching	
Patient Examination	
Question 3: is the pain located in an area where the exam unveils	
8. Hypoesthesia to touch?<br>9. Hypoesthesia to pinprick?	
Question 4: is the pain provoked or increased by	
10. Brushing?	
Yes=1 / No= 0	Patient's Score: /10

Scores ≥ 4/10 indicate neuropathic pain
